# Significant Role of Dicer and miR-223 in Adipose Tissue of Polycystic Ovary Syndrome Patients

**DOI:** 10.1155/2019/9193236

**Published:** 2019-11-11

**Authors:** Lang Qin, Jiao Chen, Li Tang, Tao Zuo, Hanxiao Chen, Rui Gao, Wenming Xu

**Affiliations:** ^1^Reproductive Medical Center, Department of Obstetrics and Gynecology, West China Second University Hospital, Sichuan University, Chengdu 610041, China; ^2^Key Laboratory of Birth Defects and Related Diseases of Women and Children of Ministry of Education, West China Second University Hospital, Sichuan University, Chengdu 610041, China; ^3^Department of Ultrasonography, West China Second University Hospital, Sichuan University, Chengdu, Sichuan, China; ^4^Reproductive Endocrinology and Regulation Laboratory, West China Second University Hospital, Sichuan University, Chengdu 610041, China; ^5^Sichuan University-The Chinese University of Hong Kong Joint Laboratory for Reproductive Medicine, Sichuan University, Chengdu 610041, China; ^6^West China School of Medicine, Sichuan University, Chengdu, Sichuan 610041, China; ^7^Department of Obstetrics and Gynecology, West China Second University Hospital, Sichuan University, Chengdu 610041, China

## Abstract

Polycystic ovary syndrome (PCOS) is a chronic metabolic disease that is associated with obesity and adipose tissue dysfunction. This study aimed to explore the roles of Dicer (an enzyme that processes primary microRNAs) and microRNAs in PCOS. Protein levels were detected by western blotting, and mRNA and microRNA levels were detected by RT-PCR. Dicer-deficient pre-adipocytes were established by lentiviral transfection, and an miR-223 mimic and miR-223 inhibitor were used to overexpress and inhibit miR-223, respectively. 3T3-L1 cells were induced to differentiate into mature adipocytes by IBMX, insulin, and dexamethasone. The degree of differentiation was determined by oil red O staining. An insulin resistance model was established by exposing mature adipocytes to excessive glucose and insulin. The protein levels of Dicer and Ago2 in adipose tissues of PCOS patients were significantly lower than those in control females. A Dicer-deficient 3T3-L1 cell model was successfully established, whose proliferation was inhibited significantly. Insulin-resistant mature adipocytes expressed significantly less Dicer protein than control cells. The differentiation of Dicer-deficient 3T3-L1 cells and their expression of miR-223 and marker genes associated with adipose differentiation were reduced significantly. Furthermore, 3T3-L1 cells showed a weaker ability to develop into mature adipocytes when miR-223 expression was inhibited. An miR-223 mimic was used to recover the differentiation block induced by Dicer deficiency. This rescued the expression of genes associated with adipose differentiation, although the differentiation block was not efficiently rescued. It is concluded that insulin resistance may contribute to the decreased levels of Dicer protein in adipose tissue of PCOS patients. This suggests that dysfunction of Dicer plays a significant role in obesity of PCOS patients. miR-223 is a key factor in Dicer-regulated adipose differentiation, and other microRNAs may be involved in the process.

## 1. Introduction

Polycystic ovary syndrome (PCOS) is a common endocrine disease in women. It is characterized by infrequent menstruation or amenorrhea, rare ovulation or anovulation, infertility, hirsutism and acne. It is often accompanied by hyperandrogenemia, insulin resistance, obesity, and other diseases [1–3]. Between 5% and 10% of women of childbearing age are infertile as a result of PCOS, of which about 50% are obese and show insulin resistance. Obesity in these patients significantly increases the risk of infertility [4]. MicroRNAs (miRNAs) are noncoding single-stranded RNA molecules of 22–24 nucleotides, which bind to the 3′-noncoding region of target mRNAs to inhibit their translation or initiate their degradation. Through the post-transcriptional regulation of target genes [5], they are also important players in the physiological regulation of PCOS [6–9]. The maturation of miRNAs has three stages: the transcription of an endogenous miRNA gene to generate a pri-miRNA, processing of the pri-miRNA into a pre-miRNA, and cleaving of the pre-miRNA by Dicer to form the mature single-stranded miRNA molecule. Therefore, Dicer is an important limiting factor of miRNA functions [5].

Dicer and many miRNAs are involved in adipose tissue differentiation, lipid droplet recruitment, and the occurrence of obesity [10, 11], and many of these miRNAs are abnormally expressed in patients with PCOS [12]. The amount of adipose tissue in Dicer-deficient mice is significantly decreased [13], while the preadipocytes of Dicer-deficient mice show obviously disrupted adipose differentiation [14]. miRNAs are involved in numerous physiological activities of adipose tissue. Some miRNAs have conserved regulatory roles, while the function of some miRNAs will vary because of species differences [15, 16]. For example, Let-7 promotes fat formation in preadipocytes and stem cells, but plays the opposite role in obese adipose tissue and adipocytes [16]. Considering that obesity plays a significant role in the etiology of PCOS, it is important to study the role of Dicer and related miRNAs in the adipose tissue of patients with PCOS from the perspectives of the mechanism-of-action as well as their therapeutic potential.

## 2. Materials and Methods

### 2.1. Subjects and Adipose Tissue Collection

This study was approved by the Ethics Committee of West China Second University Hospital of Sichuan University. Informed consent was obtained from each subject before the study. The study was performed in accordance with the 2002 International Ethical Guidelines for Biomedical Research Involving Human Subjects of the Council for International Organizations of Medical Sciences (CIOMS). Twelve PCOS cases were selected according to the Rotterdam diagnostic criteria published in 2003. Patients with common endocrine diseases were excluded. Ten control subjects were recruited, and those with acute salpingitis or endometriosis were excluded. All adipose tissue samples were obtained from the abdominal omentum. Adipose tissue was collected at the beginning of each operation (within 30 min) into sterile 50-mL conical tubes, immediately flash frozen in liquid nitrogen, and then stored at −80°C for later experiments. A total of 0.3 g fat tissue was placed in 3 mL of a 0.9% saline solution for homogenization on ice to prepare a 10% tissue homogenate.

For IVF patients, follicular fluid (FF) which was not contaminated by visible blood or aspiration buffer was aspirated and pooled at the time of oocytes retrieval for each patient. After washing with Hanks' balanced salt solution, cells were resuspended with PBS and both the granulosa cell and follicular fluid was collected in pellets and suspension, respectively.

Samples from 7 PCOS subjects and 6 controls were used for the adipose Dicer expression assay. Samples from 6 control and 7 PCOS subjects were used to evaluate miR-15b in granulosa cells, and 10 control and PCOS subjects were used for miR-15b analysis in follicular fluid. The anthropometric and biochemical parameters were shown in a new table (Supplemental [Supplementary-material supplementary-material-1]).

### 2.2. Adipogenic Induction of 3T3-L1 Cells

3T3-L1 cells from the cell bank of the Chinese Academy of Sciences were cultured in high glucose Dulbecco's modified Eagle's medium (DMEM; Gibco, USA) containing 10% fetal bovine serum (FBS; Gibco) in a 5% CO_2_ incubator (Thermo Fisher, USA) at 37°C. A Petri dish was coated with polylysine, and 3T3-L1 cells were cultured to the fusion stage after lamination. Two days after fusion (day 0), induction medium containing 0.5 mmol/L IBMX, 10 *μ*g/mL insulin, and 1 *μ*mol/L dexamethasone was added, and the whole induction process was followed as per reference [9] . On the second day, the medium was replaced with insulin-only medium. On the fourth day, the medium was replaced with nonsupplemented medium. The culture medium was changed every 2 days, and the differentiation was complete in approximately 10 days.

### 2.3. Oil Red O Staining

After careful removal of the culture medium, adipocytes were gently rinsed with PBS and then fixed for at least 30 min with 4% paraformaldehyde. An oil red O staining solution (Sigma, USA) was prepared by thorough mixing, filtering through paper filter, and resting at room temperature for 10 min. After fixation, cells were rinsed with 60% isopropanol and then stained in the oil red O working solution for 10–60 min. The oil red O solution was then removed, and the cells were rinsed with 60% isopropanol. Glycerin-gelatin were used to mount cells on coverslips, followed by examination under a light microscope.

### 2.4. Cell Proliferation Analysis

Cells in the logarithmic phase of growth were collected and seeded at 3000–5000 cells per well in 96-well plates. Twenty microliters of an MTT solution (5 mg/mL, 0.5% MTT) was added to each well, and the cells were incubated for 4 hours. The culture medium was then carefully removed, and 150 *μ*L dimethyl sulfoxide was added and mixed. Absorbance was detected at OD = 490 nm.

#### 2.5. Lentivirus Production and Establishment of a Dicer Knockdown Cell Line.

The HEK293T cell line was cultured in DMEM supplemented with 10% FBS and 1% antibiotics (100 IU/ml penicillin and 100 *μ*g/ml streptomycin) at 37°C in the humidified incubator with 5% CO_2_. Dicer knockdown in 3T3-L1 cells was achieved by lentiviral shRNAs. Lentiviral particles were produced from HEK293T cells cultured in 10-cm dishes, which were cotransfected with 4.5 *µ*g packaging plasmid psPAX2 (12260; Addgene), 1.5 *µ*g envelope plasmid pMD2.G (12259; Addgene), and 6 *µ*g pSicoR human Dicer3 or scramble shRNAs (14765, 1864-LV; Addgene) using Lipofectamine 3000 (Invitrogen). After 24 and 48 h, the culture supernatants containing viral particles were harvested twice, filtered through 0.45-*µ*m filters, and pelleted at 100,000 rcf for 2 min at 4°C. 3T3-L1 cells were selected by incubation with 1 *µ*g/ml puromycin (Invitrogen) for 72 h after transfection of sh-Dicer or sh-scr lentiviral particles to obtain lentivirus-infected stable clones (sh-Dicer and sh-scr 3T3-L1 cells, respectively) for 3 days.

### 2.6. Real-Time Fluorescence Quantitative PCR (RT-PCR)

RNA was extracted using Trizol (Life Technologies, USA), and its concentration was determined. cDNA was generated using a reverse transcription kit (Takara, Japan). SYBR Green (Life Technologies) was used for fluorescence quantification. PCR primers used were as follows. PPAR*γ*; sense: TCGCTGATGCACTGCCTATG; antisense: GAGAGGTCCACAGAGCTGATT; C/EBP*α*; sense: CAAGAACAGCAACGAGTACCG; antisense: GTCACTGGTCAACTCCAGCAC. aP2: antisense: AAGGTGAAGAGCATCATA. Quantitative real-time PCR was performed using an Applied Biosystems 7500 Sequence Detection System with SYBR green chemistry. GAPDH were used as endogenous controls for mRNA. Data were analyzed by the 2^−ΔΔ^ Ct method.

### 2.7. miRNA Gene Expression Assay

Total RNA was isolated using TRIzol (Invitrogen), and cDNA was synthesized by miRNA-specific reverse transcription primers for miRNA (Ribo Bio, Guangzhou, China) or. The cDNA of miRNA was amplified by specific forward primers and a universal reverse primer (Ribo Bio). Quantitative real-time PCR was performed using an Applied Biosystems 7500 Sequence Detection System with SYBR green chemistry. U6 and GAPDH were used as endogenous controls for miRNAs and mRNA, respectively. Data were analyzed by the 2^−^^ΔΔ^ Ct method.

### 2.8. miRNA Transfection Assays

miR-223 mimic or negative control (Ribo Bio) were transfected at 100 ng/ml using Lipofectamine 3000, in accordance with the manufacturer's protocol. After 48 hours of transfection, western blotting was conducted (Promega). All transfections and assays were repeated three times.

### 2.9. Western Blotting

Tissue samples and cells were lysed using RIPA lysis buffer and centrifuged at 12,000 × g for 20 min to collect the supernatant without lipid. Protein concentration was measured using the BCA protein assay (Thermo Scientific, MA, USA). 20 *μ*g protein was loaded onto an SDS-polyacrylamide gel, electrophoresed, and transferred to polyvinylidene difluoride membranes (PVDF, Minipore, MA, USA). The membranes were blocked with 5% nonfat milk at room temperature and then incubated with primary antibodies at 4°C for overnight. Each membrane was washed with TBST three times for 15 min followed by incubation with an HRP-conjugated secondary antibody (Zhongshan Jiangqiao, Beijing, China) at room temperature for 1 h. Finally, each membrane was developed using an enhanced chemiluminescence (ECL) detection kit (Minipore, MA, USA) and visualized using X-OMAT BT film (Carestream, Toronto, Canada). The antibody against Dicer1 was purchased from Shanghai Shangong (China). Antibodies against PPAR, C/EBP, and aP2 were obtained from Proteintech Group (Chicago, USA), and an antibody against Ago2 was purchased from CST (Boston, USA). *β*-Actin and tubulin (Zenbio Biotech Co., Ltd, Chengdu) were used as internal controls.

### 2.10. Statistical Analysis

Data are presented as mean + SD. Normality of data distribution was tested with the use of the Shapiro–Wilk test. Two groups were assessed using the Student *t* test. Data including more than two groups were assessed by one-way analysis of variance (with Tukey test for post hoc analysis). Data were analyzed with the use of Graphpad Prism 5 software, and *P* < 0.05 was considered as significant difference.

## 3. Results

### 3.1. Expression of Dicer in Adipose Tissue of PCOS Patients

**Figure 1:**
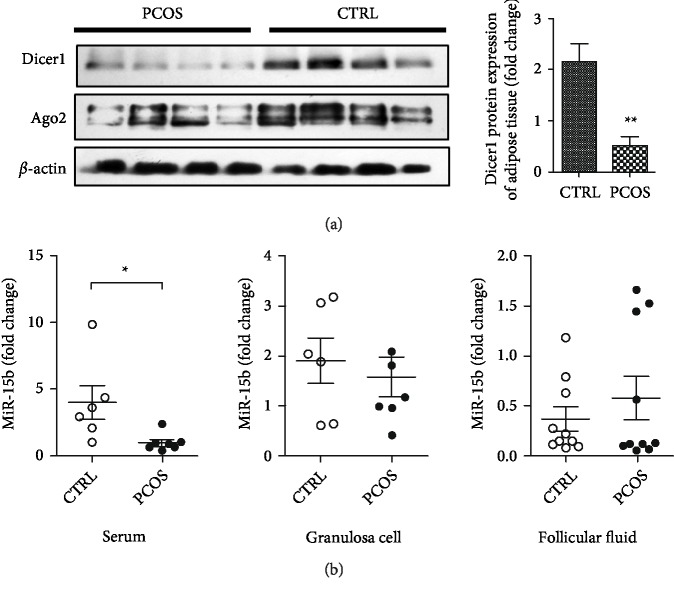
Expression of Dicer and related miRNAs in adipose tissue and serum of PCOS patients. (a) Western blots result showing reduced Dicer expression in adipose tissue of PCOS patients and control subjects (*N* = 4 for control; *N* = 4 for patients). (b) Real-time PCR showing reduced expression of miR-15b in serum of PCOS patients (*N* = 6 for control; *N* = 7 for patients), while no significant change was found in granulosa cells (*N* = 6 for control; *N* = 7 for patients) or follicular fluid (*N* = 10 for control; *N* = 10 for patients) of the patients. ∗*p* < 0.05, ∗∗*p* < 0.01.

Four samples of subcutaneous omental adipose tissue were collected from PCOS patients and control subjects. Western blotting showed that Dicer and Ago2 protein levels in adipose tissue of PCOS patients were significantly lower than those in controls (Figure 1(a)). Because our previous study had shown that Dicer regulates the expression of miRNA, and one miRNA, miR-15b, was significantly dysregulated [17], we detected miR-15b expression. The results showed that miR-15b was downregulated in the serum of PCOS patients, while no significant change of miRNA expression was observed in granulosa cells or follicular fluid of PCOS patients (Figure 1(b)). These results showed that dysregulated expression of Dicer and a related miRNA is a significant feature of PCOS.

### 3.2. Dicer Expression Is Downregulated in the Insulin-Resistant Model

**Figure 2:**
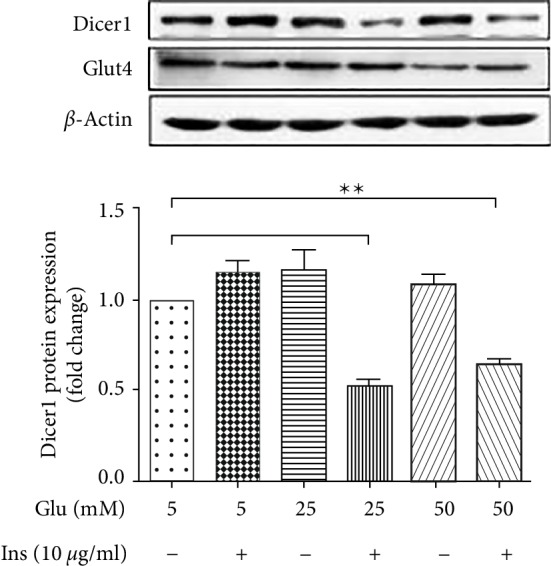
Expression of Dicer is significantly reduced in the insulin resistance model. Western blot results of Dicer1 expression in the insulin resistance model. GLU4 was used as a marker for successful establishment of the model.

It has been shown that insulin resistance is one of the major features associated with PCOS. Because the expression of Dicer was downregulated in adipose of PCOS patients, we determined whether the insulin resistance led to altered expression of Dicer. To generate an insulin resistance model, mature adipocytes were cultured in low glucose medium (Cat. No: 10567-014, Thermo Fisher) for 2 days to adapt to a low glucose environment. Cells were then cultured in high glucose (25 mmol/L) and high insulin (10 *μ*g/mL) medium for 48 hours, washed with PBS three times, and then cultured in high glucose and high insulin (10 *μ*mol) medium for 48 h. Cells were then incubated in KPBH buffer containing 1% BSA and low concentration insulin (1 *μ*g/mL) for 30 min [17]. Six experimental groups were prepared according to the glucose concentration (5, 25, and 50 mM) and the presence or absence of insulin. Cells were induced according to the above scheme, and protein was extracted. The levels of Dicer and GLUT4 proteins were then measured. GLUT4 is a glucose transporter and important candidate gene for insulin resistance. GLUT4 levels decreased with the increase in glucose concentration. The increasing glucose concentration promoted the expression of Dicer, but when high glucose and high insulin led to insulin resistance, the expression of Dicer was significantly inhibited (Figure 2).

### 3.3. Dicer-Deficient Preadipocytes Show Reduced Proliferation and Differentiation

**Figure 3:**
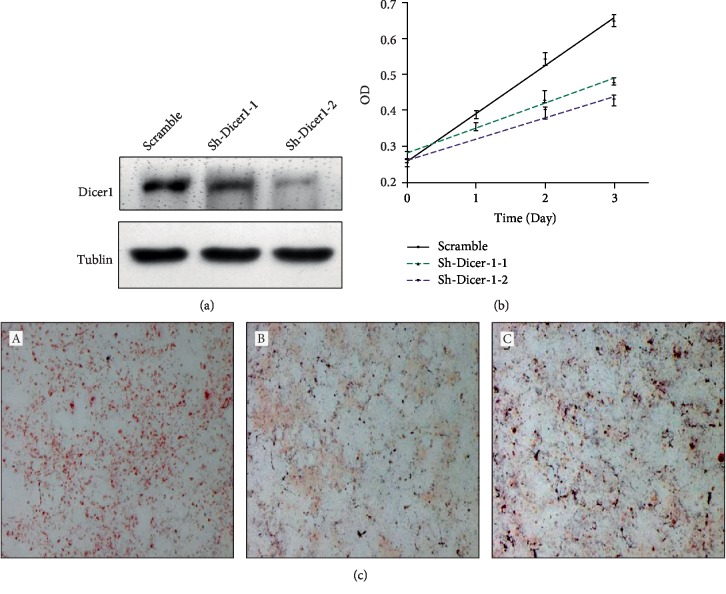
Knockdown of Dicer reduces the proliferation and differentiation of adipose cell line 3T3-L1. (a) Western blot results showing that Dicer expression was reduced after knockdown of Dicer1. (b) Cell proliferation curve indicating that knockdown of Dicer1 reduced proliferation at day 2 and 3. (c) Oil red O staining showing the difference in adipogenic differentiation between control and Dicer-deficient cell lines.

A lentivirus-transfected Dicer shRNA plasmid was used to establish a Dicer-deficient adipose precursor (3T3-L1) cell line. The expression of Dicer in the Dicer shRNA-transfected cell line was significantly decreased, indicating that the Dicer-deficient cell line was successfully established (Figure 3(a)). In addition, the proliferation of the Dicer-deficient cell line was significantly decreased compared with that of control 3T3-L1 cells (Figure 3(b)). The effect of Dicer knockdown on differentiation was then examined. 3T3-L1 adipocytes were induced to differentiate into adipocytes by IBMX, insulin, and dexamethasone, and the degree of differentiation was identified by oil red O staining after 8 days. Most precursor cells had successfully differentiated into mature adipocytes as reflected by positive staining (*A*).

Dicer-deficient (Sh-Dicer-2) and control (Scramble) cell lines were prepared. After adipocyte differentiation, oil red O staining was used to identify the effects on differentiation. The results showed that the ability of 3T3-L1 cells to differentiate was significantly decreased after Dicer knockdown (Figure 3(c)).

### 3.4. Expression of Differentiation-Related Marker Genes and Dicer during the Differentiation Process

**Figure 4:**
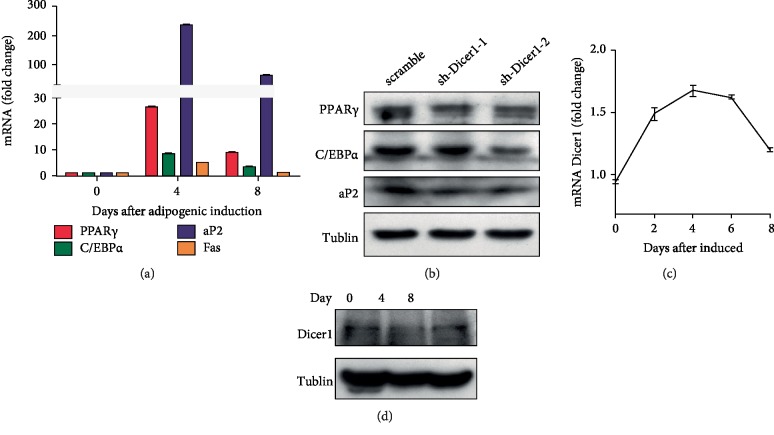
Knockdown of Dicer reduces adipogenesis marker gene expression during the differentiation process. (a) Expression of adipogenesis genes, including PPAR, CEBP, aP2, and Fas during the differentiation process. (b) Western blot results of adipogenesis marker genes after Dicer knockdown. (c) mRNA expression of Dicer during the differentiation process. Real-time PCR results of Dicer transcript during the differentiation process of adipogenesis. (d) Western blot results show the expression change of Dicer during the differentiation process.

Cells were collected on day 0, 4, and 8, and mRNA was extracted to determine the expression of PPAR*γ*, C/EBP*α*, aP2, Fas, and Dicer. PPAR*γ*, C/EBP*α*, aP2, and Fas are important for adipocyte differentiation. Their expression increased sharply during differentiation and then decreased (Figure 4(a)) at the later stage of differentiation. The expression of both mature adipocyte marker genes (PPAR*γ* and C/EBP*α*) was significantly lower in Sh-Dicer-2 cells compared with Scramble cells after differentiation (Figure 4(b)). Furthermore, there was only a slight increase in Dicer expression during adipocyte differentiation, but it was not significant (Figures 4(b) and 4(c)). Real-time PCR shows that Dicer transcript expression is increased first, then back to normal during the whole induction process (Figure 4(c)). Western blotting further showed that the expression of Dicer1 was reduced on day 4, while back to normal after day 8 (Figure 4). Together, the results indicate that Dicer plays an important role in regulation of key adipose genes during the adipose differentiation process.

### 3.5. Effect of miR-223 Deficiency on Adipogenic Differentiation

**Figure 5:**
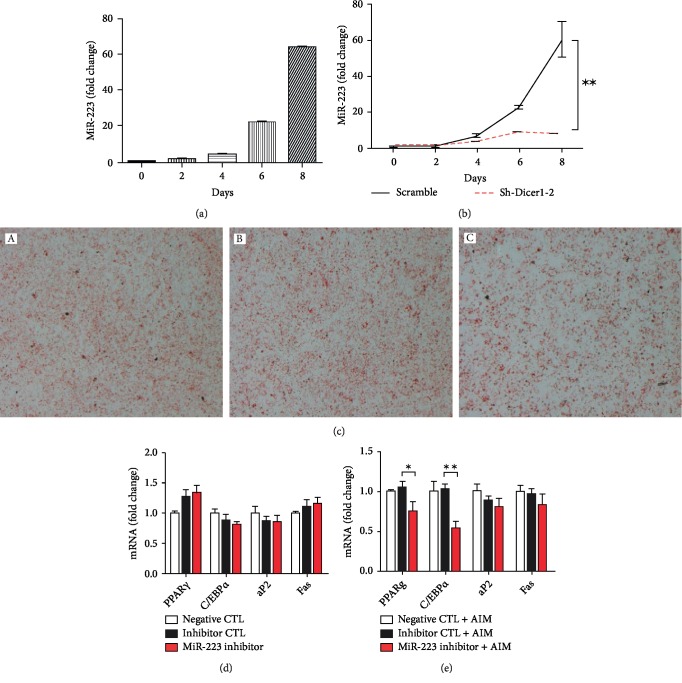
Dicer regulates miR-223 that plays a critical role in the adipose differentiation process. (a) Expression of miR-223 during the adipose differentiation process. (b) Expression of miR-223 after knockdown of Dicer1. Significantly reduced expression of miR-223 was found after knockdown of Dicer1. (c) Oil red O staining showed reduced differentiation after applying the miR-223 inhibitor to 3T3-L1 cells. (d) No significant change of adipogenesis marker genes was observed after knockdown of Dicer1 genes. (e) Significantly reduced expression of aP2, PPAR, and other genes was observed after knockdown of Dicer and in the induced differentiation model.

We first examined the expression change of miR-223 during adipose differentiation. The expression of miR-223 was upregulated during adipose differentiation (Figure 5(a)). Because it has been shown that Dicer affects most miRNA processing, we checked the effect of Dicer on the expression of miR-223. The results showed that knockdown of Dicer decreased the expression of miR-223 (Figure 5(b)). We then confirmed the effect of miR-223 on the expression of differentiation-related marker genes. miRNA inhibitors are commonly used to target specific miRNAs in cells. 3T3-L1 cells were transfected with an miR-223 inhibitor and then differentiated. After differentiation, oil red O staining was used to determine the effect. The differentiation of 3T3-L1 adipose precursor cells transfected with the inhibitor was weaker than that of the negative control and inhibitor control cells (Figure 5(c)). The expression of adipogenic differentiation marker genes was determined at 48 h after inhibitor transfection. There was no significant change in expression of these marker genes after inhibiting the expression of miR-223 (Figure 5(d)). However, after the induction process, the expression of C/EBP*α* and aP2 in inhibitor-transfected cells was significantly lower than that in negative control and inhibitor control cells, especially for aP2 (Figure 5(e)). The reduced expression of aP2 in inhibitor-transfected cells indicates that miR-223 plays a significant role in adipogenesis, at least in our model.

### 3.6. MiR-223 Rescues the Phenotype and Gene Expression of Disrupted Adipogenic Differentiation Caused by Dicer Deficiency

**Figure 6:**
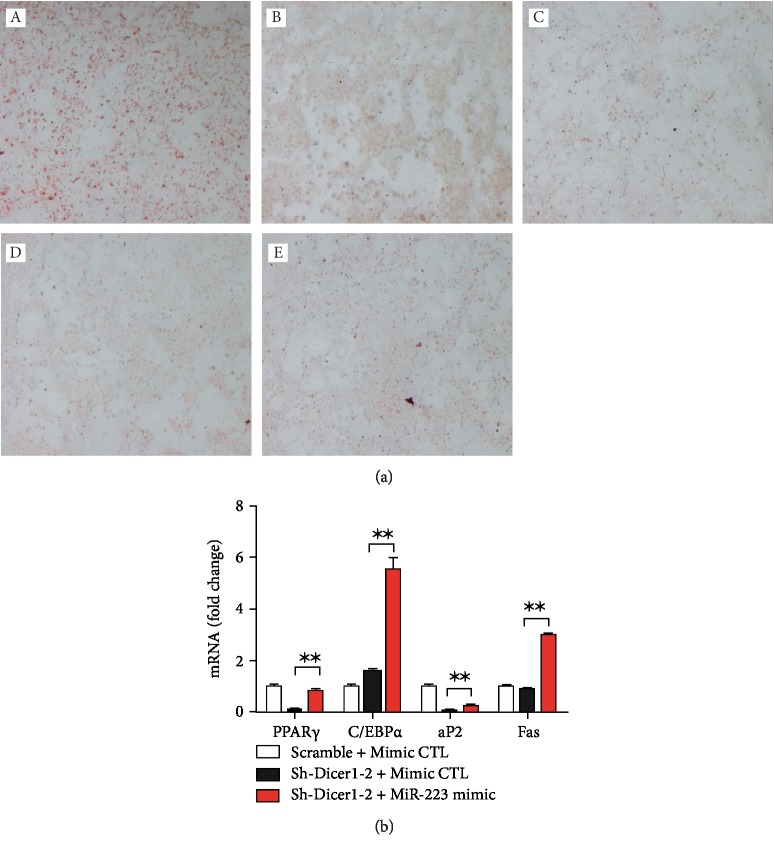
miR-223 overexpression partially rescues differentiation defects and related gene expression during the adipose differentiation process. (a) Effect of miR-223 overexpression on the rescue of disrupted adipogenic differentiation caused by Dicer deficiency. Oil red O staining was used to determine the differentiation status. (b) Effect of miR-223 overexpression on the low expression of adipogenic differentiation-related genes induced by Dicer deficiency. ∗*p* < 0.05, ∗∗*p* < 0.01.

To further confirm the role of miR-223 in Dicer-mediated adipose differentiation, three experimental groups of 3T3-L1 cells were prepared: Scramble + mimic control, Sh-Dicer-2+ mimic control, and Sh-Dicer-2+ mimic miR-223. An miRNA mimic is a chemically synthesized miRNA analogue that simulates high level expression of an endogenous mature miRNA in cells to enhance the function of the miRNA. The above three experimental groups of 3T3-L1 cells were prepared to induce differentiation into mature adipocytes, and the degree of differentiation was identified by oil red O staining. Addition of the mimic increased the degree of differentiation of Dicer-deficient cells, although the effect was not significant (Figure 6(a)). However, the expression of adipose differentiation marker genes, including PPAR*γ*, C/EBP*α*, and Fas, was significantly altered during cell differentiation and significantly increased in Dicer-deficient cells treated with the miR-223 mimic (Figure 6(b)). Together, these findings identified miR-223 as a key miRNA regulated by Dicer during adipose differentiation.

## 4. Discussion

Our study indicates that insulin resistance may contribute to the decreased levels of Dicer protein in adipose tissue of PCOS patients. This suggests that dysfunction of Dicer plays a significant role in obesity of PCOS patients. In addition, miR-223 was identified as a key factor in Dicer-regulated adipose differentiation, although other microRNAs may be involved in the process.

Knockdown of Dicer in 3T3-L1 cells clearly disrupted differentiation, indicating that Dicer plays a key role in adipogenic differentiation [18]. Moreover, Dicer had a significant regulatory effect on the expression of adipogenesis-related genes, including PPAR*γ*, C/EBP*α*, and Fas, and knockdown of Dicer effectively decreased the expression of these adipose differentiation-related genes. We believe that Dicer-related regulation is likely to play a direct role in the differentiation process, and the effect may be mediated through one or more miRNAs.

miRNAs generally inhibit target mRNAs and directly act on and downregulate PPAR*γ*, C/EBP*α*, and other genes [19]. Therefore, if miRNAs mediate the effect of Dicer on PPAR*γ*, C/EBP*α*, and other genes, identification of the key miRNAs regulated by Dicer is of significance. miR-223 promotes adipogenic differentiation of 3T3-L1 cells [19], and it has a positive regulatory effect on PPAR*γ* and other genes. We therefore hypothesized that miR-223 is a key miRNA connecting Dicer with PPAR*γ* and C/EBP*α*. Our results showed that miR-223 was highly expressed during the process of adipogenic differentiation, and that the expression of miR-223 in Dicer knockdown cells was greatly reduced, which support our hypothesis. Moreover, inhibition of miR-223 expression compromised adipogenic differentiation, and the lack of miR-223 during induction resulted in low expression of C/EBP*α* and aP2. Furthermore, miR-223 had a direct and significant regulatory effect on C/EBP*α* and aP2 expression. The miR-223 mimic was used to restore the differentiation process inhibited by Dicer knockdown. We found that the expression of genes, such as PPAR*γ* and C/EBP*α*, was increased significantly, and the degree of differentiation tended to increase compared with the degree of differentiation of control progenitor cells. Therefore, miR-223 may play a role in the regulation of C/EBP*α* and aP2 by Dicer, although other miRNAs are also possibly involved in the regulation of Dicer, PPAR*γ*, C/EBP*α*, and other genes. Thus, the regulatory mechanism of Dicer requires further investigation.

Insulin resistance is a typical symptom of PCOS [20–22]. We established an insulin resistance model at the cellular level and found that the expression of Dicer was significantly inhibited by insulin resistance. Clinical samples also showed that Dicer expression was significantly decreased in adipose tissue of PCOS patients, indicating that insulin resistance is an important contributing factor affecting the expression of Dicer in PCOS.

Decreased levels of Dicer protein were found in adipose tissue of PCOS patients. Cell culture studies indicate that Dicer plays an important role in the differentiation of 3T3-L1 cells into mature adipocytes. miR-223 regulates the differentiation of 3T3-L1 cells to a certain extent and may be one of the main mediators of Dicer-regulated differentiation of 3T3-L1 cells. Insulin resistance may induce downregulation of Dicer in adipose tissue of PCOS patients. Drugs, such as Enoxacin, have been demonstrated to enhance the activity of Dicer [23]. Interestingly, Enoxacin also reduces obesity. Therefore, the current study indicates that Dicer and miRNAs, such as miR-223, are important etiological factors in the obesity related to PCOS and provides drug targets for future research.

## Data Availability

The research article data used to support the findings of this study are available from the corresponding author upon request. Xu Wenming, Ph.D, M.D Department of Obstetrics and Gynecology, West China Second University Hospital, Sichuan University, Chengdu 610041, China xuwenming@scu.edu.cn.

## Supplementary Materials

Supplementary Materials Supplemental Table 1: clinical and biochemical characteristics of the PCOS and control groups. miRNA expression level was determined in the granulosa cell and follicular fluid from the patients. Supplemental Table 2: the oxidative stress and mRNA level were analyzed by univariate and multivariate two-way general linear regression models. When HOMA-IR was regressed with WC, the outcomes were adjusted for age as the age was different between the control and PCOS groups. When HOMA-IR was regressed with GLUT4, IRS1, T-SOD, GPx, and MDA separate, WC and age were used as co-variates to determine the associations between adipose tissue parameters and insulin resistance. *β* is the regression coefficient. ∗ indicates a significant difference were detected between the two regression coefficient values.
