# Supplementary material for: Significant Role of Dicer and miR-223 in Adipose Tissue of Polycystic Ovary Syndrome Patients
Source: Biomed Res Int. 2019 Nov 11;2019:9193236. doi: 10.1155/2019/9193236 (PMC6885226; doi:10.1155/2019/9193236)

|  | PCOS (n=6) | Control (n=7) | P value |
| --- | --- | --- | --- |
| Age (yr) | 26 | 28 | 0.000 |
| BMI (kg/m2) | 22.31±3.55 | 21.82±2.79 | 0.555 |
| WC (cm) | 83.23±9.45 | 82.77±8.35 | 0.840 |
| Testosterone (ng/ml) | 1.06±2.11 | 0.58±0.14 | 0.220 |
| TSH (nmol/L) | 2.37±1.36 | 2.11±1.90 | 0.379 |
| FPG (mmol/L） | 5.20±0.40 | 5.20±0.43 | 0.973 |
| FINS (mIU/L） | 13.26±8.46 *^b^* | 8.59±3.17 *^b^* | 0.006 |
| HOMA-IR | 3.18±2.21^c^ | 2.01±0.85^c^ | 0.009 |

**Supplimental Table 1**

Clinical and biochemical characteristics comparison of PCOS and control groups

**Abbreviations:**  BMI, body mass index; TSH, thyroid stimulating hormone; FT_3_/T_4_, free T_3_/T_4_; TC, total cholesterol; TG, triglycerides; LDL, low-density lipoprotein; HDL, high-density lipoprotein; FPG, fasting glucose. Data are means ± SD.

*^a,b，c^ P*＜0.01, PCOS *vs.* Control, by *t* test.

**Supplimental Table 2: Univariate and Multivariable model result for regression analysis**

|  | Univariate model | |  |  | Multivariate Model | | |  |
| --- | --- | --- | --- | --- | --- | --- | --- | --- |
|  | Non-PCOS |  | PCOS |  | Non-PCOS |  | PCOS |  |
|  | β | P | β | p | β | p | β | p |
| WC | 0.003 | 0.717 | 0.046 | 0.000 | 0.005* | 0.556 | 0.047* | 0.000 |
| GLUT4 | 0.203 | 0.186 | -0.475 | 0.058 | 0.358* | 0.008 | -0.363* | 0.088 |
| IRS1 | -0.159 | 0.396 | -0.753 | 0.003 | -0.072 | 0.672 | -0.530 | 0.026 |
| T-SOD | -0.004 | 0.476 | -0.027 | 0.000 | 0.003* | 0.644 | -0.019* | 0.003 |
| GPx | -0.001 | 0.759 | -0.030 | 0.000 | 0.004* | 0.347 | -0.021* | 0.001 |
| MDA | 0.029 | 0.601 | 0.147 | 0.001 | -0.013 | 0.809 | 0.079 | 0.104 |

**Abbreviations:** WC, waist circumstances; GLUT4, glucose transporter 4;IRS1, Insulin receptor substrate 1; T-SOD, Total Superoxide dismutase; GPx, glutathione peroxides; MDA, Dimethylarginine.

**Footnotes**: The oxidative stress and mRNA level were analyzed by univariate and multivariate two-way general linear regression models. When HOMA-IR was regressed with WC, the outcomes were adjusted for age as the age was different between the control and PCOS groups. When HOMA-IR was regressed with GLUT4, IRS1, T-SOD, GPx and MDA separate, WC and age were used as co-variates to determine the associations between adipose tissue parameters and insulin resistance. β is the regression coefficient. * indicates a significant difference were detected between the two regression coefficient values.

**Supplimental Figure 1.**


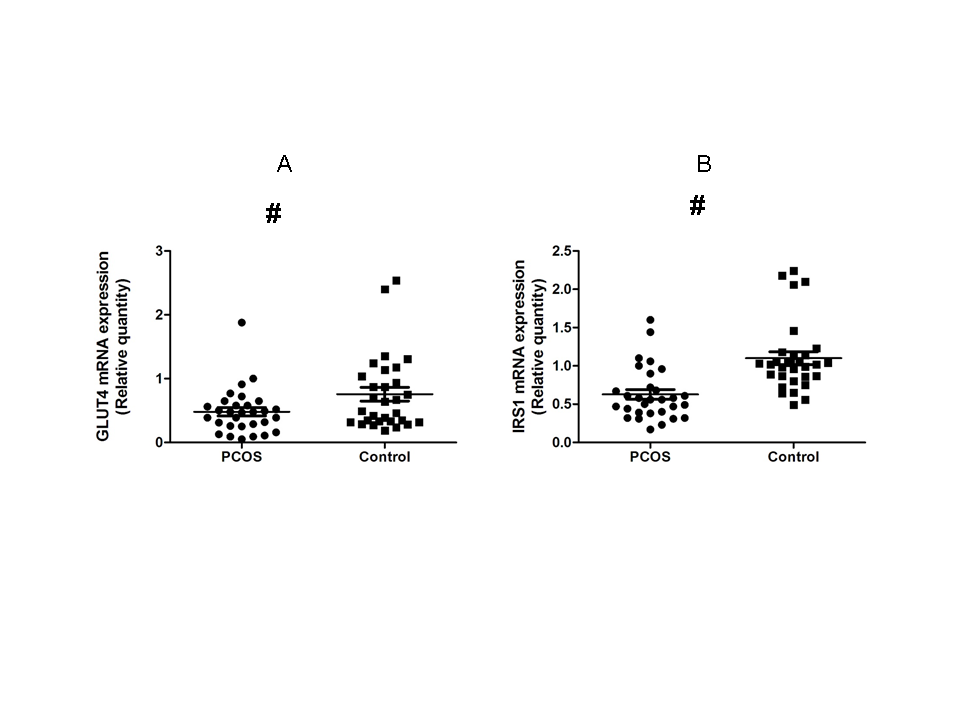


**Supplimental Figure 2**


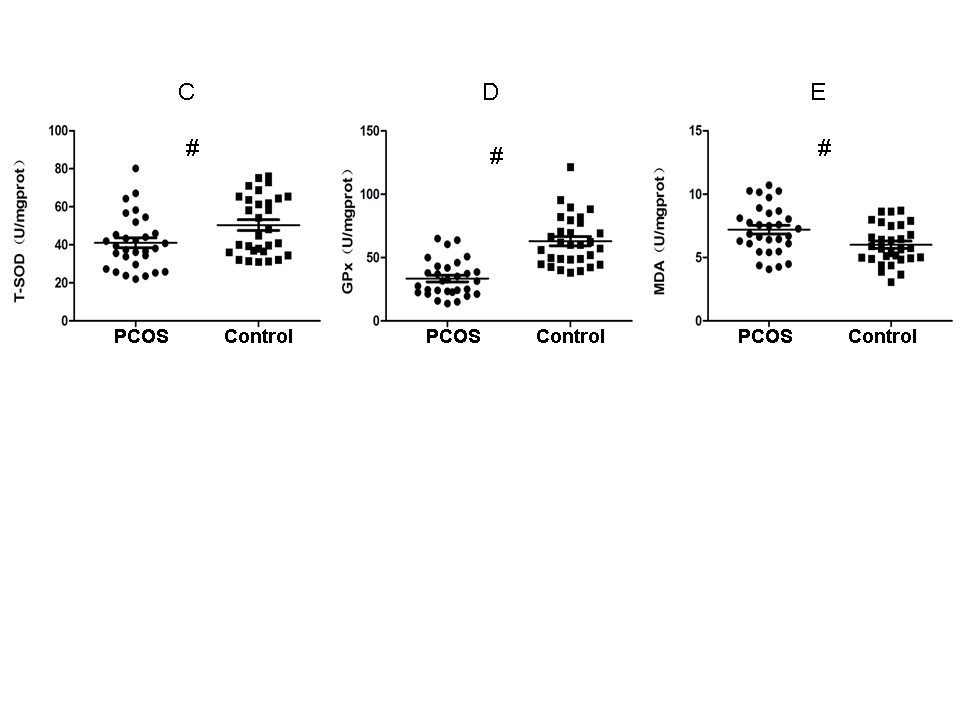

Supplement: Supplementary Materials — Supplemental Table 1: clinical and biochemical characteristics of the PCOS and control groups. miRNA expression level was determined in the granulosa cell and follicular fluid from the patients. Supplemental Table 2: the oxidative stress and mRNA level were analyzed by univariate and multivariate two-way general linear regression models. When HOMA-IR was regressed with WC, the outcomes were adjusted for age as the age was different between the control and PCOS groups. When HOMA-IR was regressed with GLUT4, IRS1, T-SOD, GPx, and MDA separate, WC and age were used as co-variates to determine the associations between adipose tissue parameters and insulin resistance. β is the regression coefficient. ∗ indicates a significant difference were detected between the two regression coefficient values. [file 9193236.f1.docx]
